# Supplementary material for: fMRIPrep Lifespan: Extending A Robust Pipeline for Functional MRI Preprocessing to Developmental Neuroimaging
Source: bioRxiv. 2025 May 18:2025.05.14.654069. Preprint. [Version 1] doi: 10.1101/2025.05.14.654069 (PMC12132292; doi:10.1101/2025.05.14.654069)
Supplement: Supplement 1 [file media-1.pdf]

Supplementary Material

fMRIPrep Lifespan: Extending A Robust Pipeline for Functional MRI Preprocessing to Developmental Neuroimaging

Mathias Goncalves [1#], Julia Moser [2#], Thomas J. Madison [2], rae McCollum [2], Jacob T. Lundquist [2], Begim Fayzullobekova [2], Lidia Hadera [2], Han H. N. Pham [2], Lucille A. Moore [2], Audrey Houghton,2,Greg Conan [2], Martin A. Styner [3], Dimitrios Alexopoulos [4], Christopher D. Smyser [4], Sally M. Stoyell [2,5], Sanju Koirala [2,5], Steven M. Nelson [2,6], Kimberly B. Weldon [2], Erik Lee [2], Robert J. M. Hermosillo [2,6], Luca Vizioli [7], Essa Yacoub [7], Gaurav H. Patel [8], Juan Sanchez [8], Kenneth Wengler [8,9], Taylor Salo [10], Theodore D. Satterthwaite [10,11], Jed T. Elison [2,5,6], Christopher J. Markiewicz [1], Russell A. Poldrack [1], Eric Feczko [2,6], Oscar Esteban [1,12], Damien A. Fair [2,5,6]

[1] Department of Psychology, Stanford University, Stanford, CA, USA
[2] Masonic Institute for the Developing Brain, University of Minnesota, Minneapolis, MN, USA
[3] Department of Psychiatry and Computer Science, University of North Carolina at Chapel Hill
[4] Department of Neurology, Washington University School of Medicine
[5] Institute of Child Development, University of Minnesota
[6] Department of Pediatrics, University of Minnesota
[7] Center for Magnetic resonance Research, University of Minnesota
[8] Department of Psychiatry, Columbia University Irving Medical Center and New York State Psychiatric Institute
[9] Departments of Psychiatry and Radiology, Icahn School of Medicine at Mount Sinai
[10] Penn Lifespan Informatics and Neuroimaging Center, University of Pennsylvania
[11] Penn-CHOP Lifespan Brain Institute
[12] Department of Radiology, Lausanne University Hospital and University of Lausanne, Lausanne, Switzerland

#These authors have contributed equally to this work

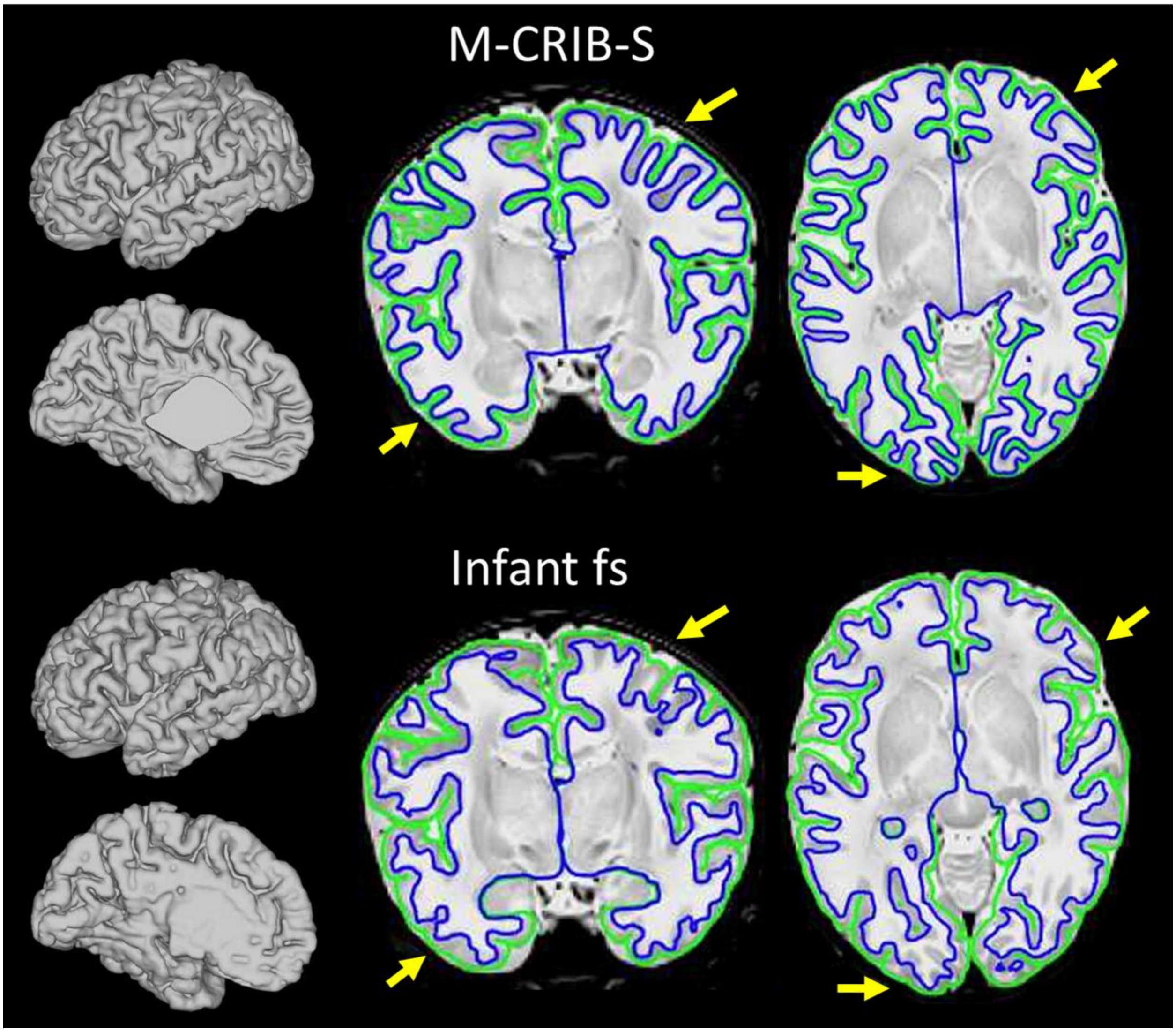

Figure S1: Example of surface reconstruction performed with M-CRIB-S and Infant fs in a one month old subject. Both methods used the same segmentation generated by BIBSNet [27] as a basis. White matter surface shows more detail with M-CRIB-S while Infant fs misses the delineation of some smaller gyri (see arrows). M-CRIB-S is optimized for T2 based surface reconstruction at very young ages.

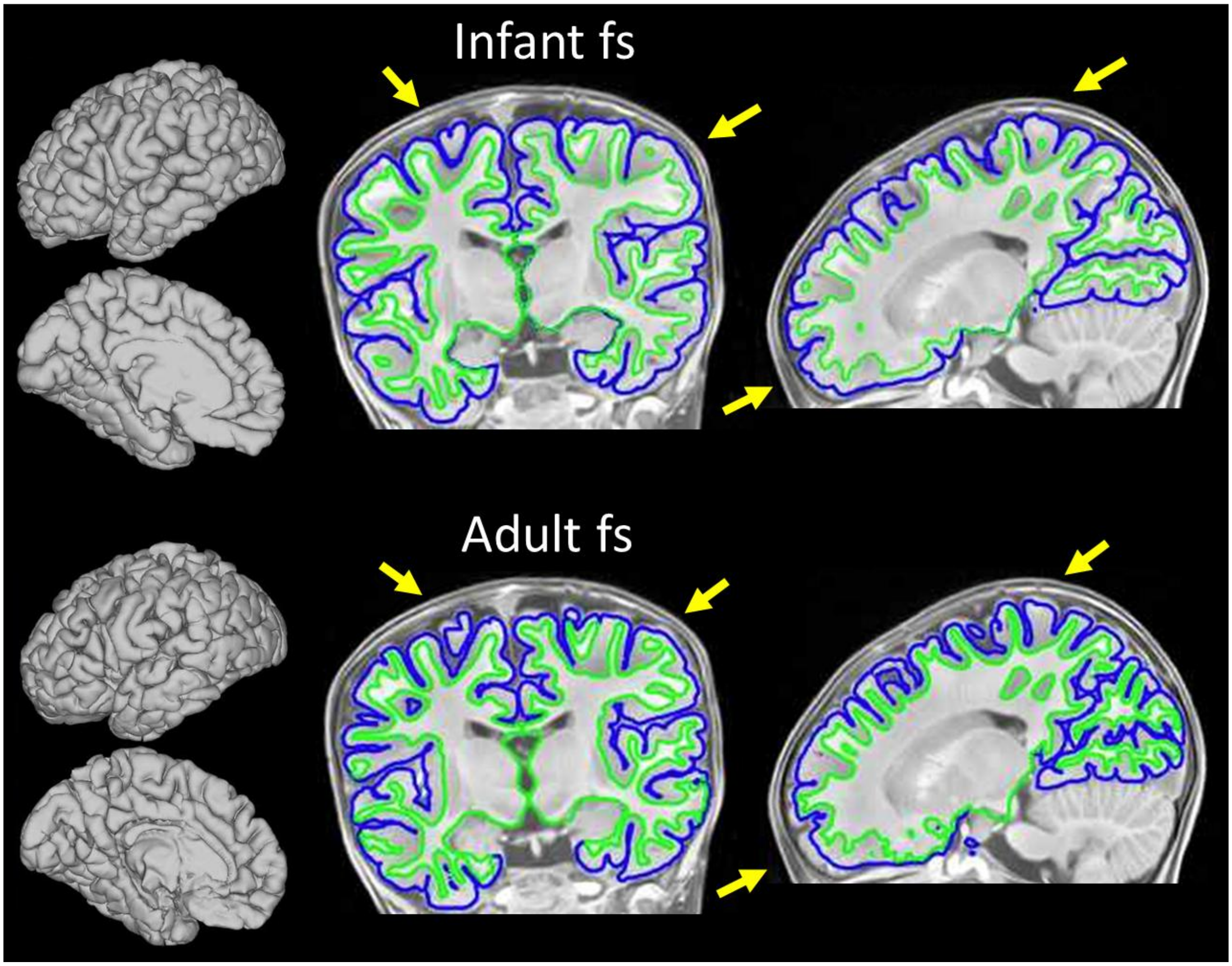

Figure S2: Example of surface reconstruction performed with Infant fs and adult Freesurfer recon-all in a 17 month old subject. Infant fs uses the segmentation and brain mask generated by BIBSNet [27] as a basis. White matter surface shows more detail in some areas with Freesurfer recon-all however in this example, the Freesurfer based brain masking leads to gray matter cutoffs, which makes the surface created by Infant fs preferable.

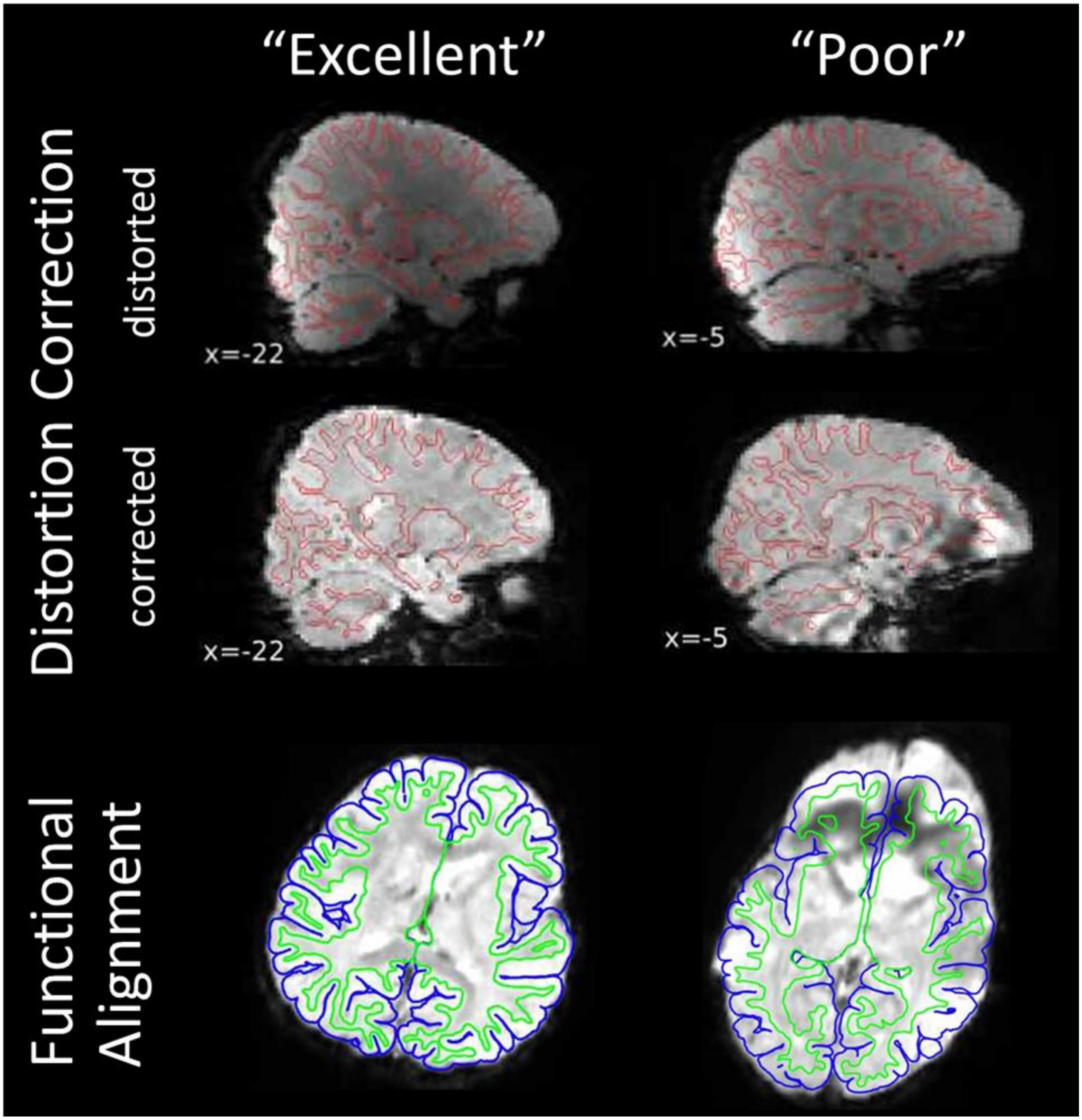

Figure S3: Example derivatives rated as "excellent" and "poor" regarding distortion correction (top) and functional alignment (bottom). Examples are a 26 month old (left) and 20 month old (right). Data quality issues in the example on the right are caused by significant motion between fieldmaps.

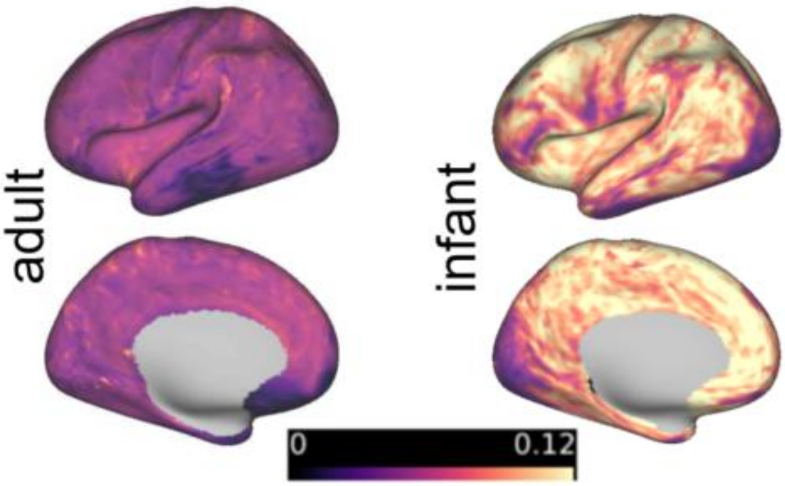

Figure S4: Example for T2 maps (modified after [20]). Maps are projected onto the cortical surface, values represent T2* relaxation time in seconds.*

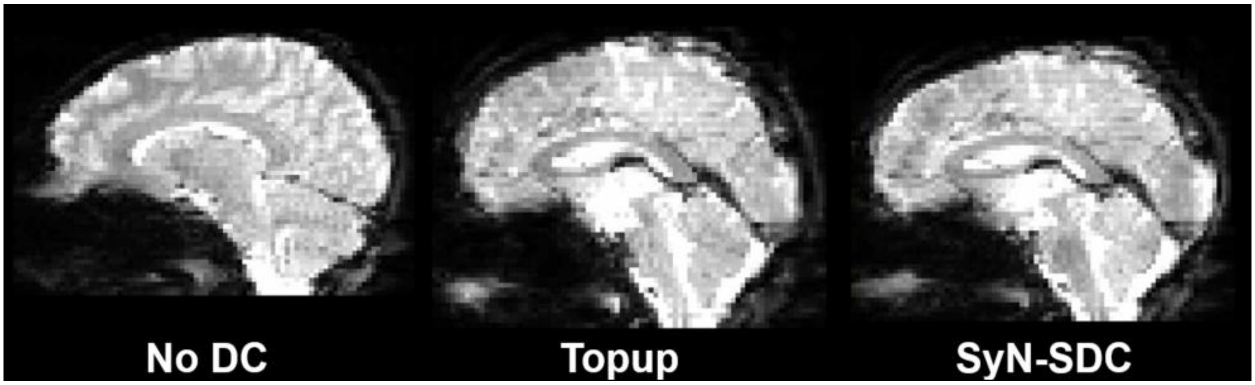

Figure S5: A) Distortion correction (DC) in example subject, comparing no DC to FSL topup and fieldmapless SyN-SDC.

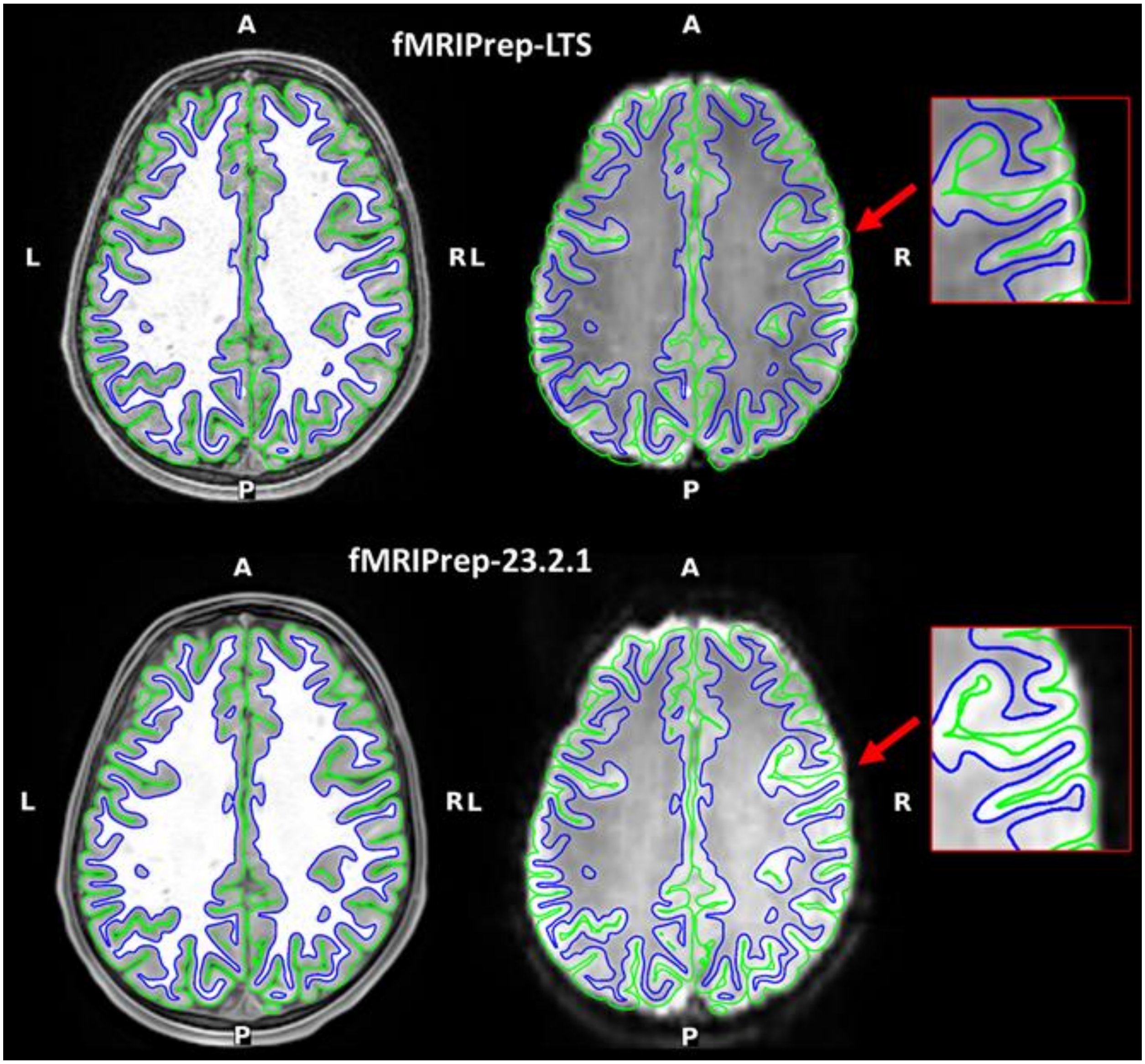

Figure S6: Comparison of registration of surface to T1w image (left) and surface to BOLD image (right) between fMRIPrep-LTS and fMRIPrep-23.2.1 for a single ABCD resting-state run (5 minutes). Changes implemented between LTS and 23.2.1 (see Suppl. Table 1) improved BOLD registration.

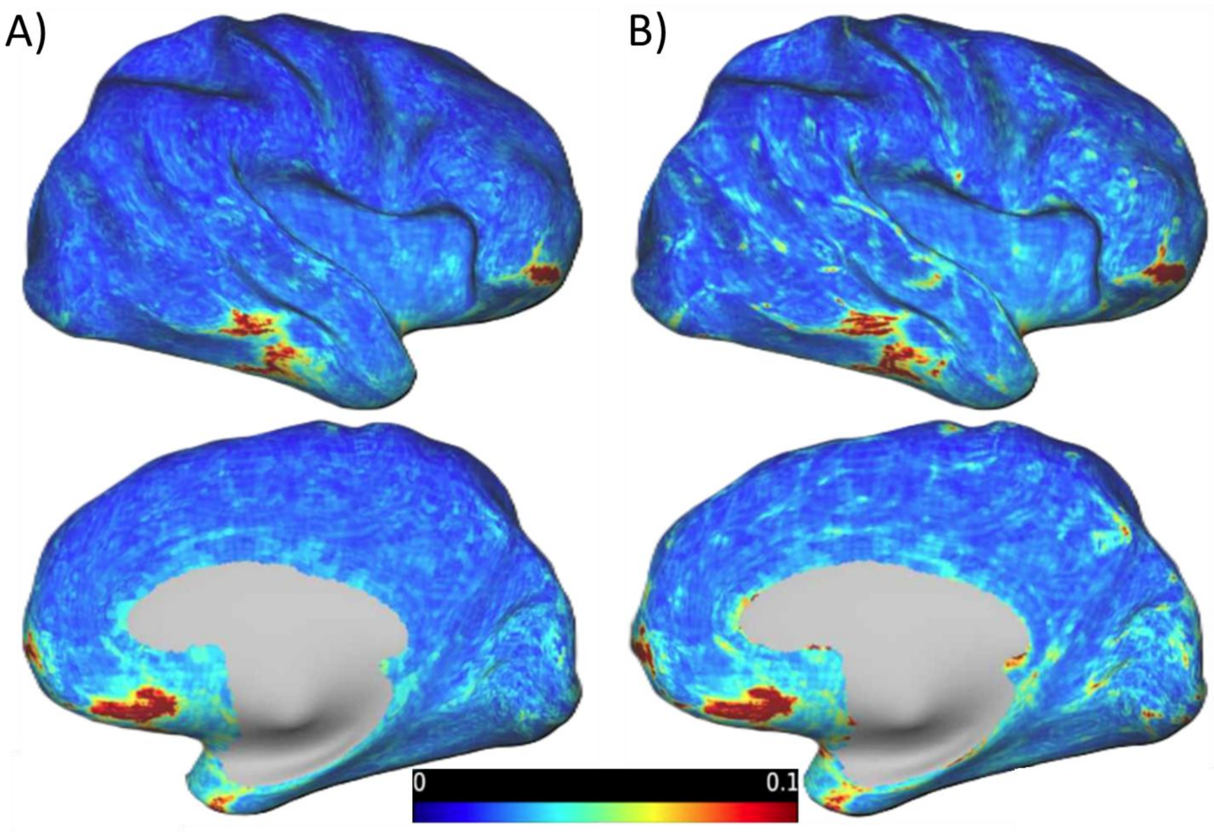

Figure S7: Comparisons of outputs with (A) and without (B) ‘good voxels’ mask applied. Figure shows coefficient of variation (SD divided by mean) for a single ABCD resting-state run (5 minutes). The ‘good voxels’ mask excludes voxels with local peaks of temporal variation from the BOLD time-series

Participant ID	Session ID	Surface reconstruction Method	Surface recon-struction		Spatial normalization		Distortion correction		Functional alignment	
			QC1	QC2	QC1	QC2	QC1	QC2	QC1	QC2
421109	1mo	mcribs	1	1	1	1	3	2	2	2
584381	1mo	mcribs	1	1	1	1	1	1	1	1
960758	2mo	mcribs	1	1	1	1	1	1	1	1
309615	3mo	mcribs	1	1	1	1	1	1	1	1
375518	4mo	infantfs	2	2	1	1	1	1	2	1
229768	5mo	infantfs	1.5	1	1	1	1	1	1	1
197622	6mo	infantfs	1	1.5	1	1	3	2	1.5	2
229768	8mo	infantfs	1	1.5	1	1	1	1	1.5	1
132476	6mo	infantfs	1	1	1	1	1	1	1.5	2
381606	5mo	infantfs	1	1.5	1.5	1	1	1	1	1
116845	9mo	infantfs	1	1.5	1	1	2	1	1.5	1
381606	11mo	infantfs	2	1	1	1	1	1	1	1
530066	13mo	infantfs	1	2	1	1	1	1	1	1
132476	15mo	adultfs	3	1	2	1	1	1	1	1
100619	17mo	infantfs	1	1.5	1	1	1	1	1	1
107842	19mo	infantfs	1	1.5	1	1	1	1	1	1
200474	21mo	infantfs	1	1.5	1	1	1	1	1	1
261266	23mo	infantfs	1	1	1	1	1	1	1	1
105040	24mo	infantfs	1	1.5	1	1	1	1	1	1
176851	20mo	infants	1	1.5	1	1	2	2.5	2	2
353374	25mo	adultfs	1	2	1	1	1	1	2	1
185373	26mo	infantfs	1	1.5	1	1	1	1	1	1
505525	26mo	infantfs	1	1.5	1	1	1	2	1.5	2
266394	27mo	infantfs	1	1.5	1	1	1.5	2	1.5	2
764612	28mo	infantfs	1	1.5	1	1	2	2.5	1.5	2.5
148796	29mo	infantfs	1	1.5	1	1	1	1	1	1
418793	32mo	infantfs	1	1	1	1	1	1	1	1
294064	34mo	infantfs	1	1.5	1	1	1.5	1	1.5	1

353374	37mo	infantfs	1	1.5	1	1	1	1	1	1
676274	43mo	infantfs	1	1	1	1	1	1	1	1

Supplementary Table 1: Quality control outcomes for all participants separated into three age bins. Results were generated by six trained raters, each output was reviewed by two independent raters. 1= rated as high quality ("excellent"); 2=rated as usable with room for improvement ("acceptable"); 3= not of acceptable quality ("poor");

**Version**	**Initial Release**	**Major Changes**
23.2.x	January 2024	Added MSMSulc-based registration to fsLR template; added option to ingress precomputed derivative files (instead of generating them as part of the fMRIPrep workflow)<br><br>SDCFlows updated to add Jacobian weighting step during unwarping, better masking of phase-difference and direct fieldmaps
23.1.x	June 2023	Overhaul of BOLD resampling workflows targeting fsLR spaces, based on HCP methods<br><br>CIFTI derivatives revised to use column-major ordering for subcortical grayordinates to match HCP 91k and 170k templates<br><br>Added T1w and T2w denoising step before surface reconstruction
23.0.x	March 2023	CIFTI dense scalar format output of FreeSurfer morphometric derivatives (curvature, cortical thickness, sulcal convexity) in fsLR space<br><br>Optional "goodvoxels" masking adapted from HCP methods<br><br>Addition of T2w volume derivatives in subject T1w space
22.1.x	December 2022	Head motion correction transforms and reference volumes added to functional derivatives<br><br>GIFTI format output of FreeSurfer morphometric derivatives<br><br>SDCFlows updated to 2.2.x, focusing on robustness of PEPolar workflow
22.0.x	July 2022	Move to FreeSurfer 7 (replacing 6.0.0)

		Estimated T2* maps and (in QA report) T2* histograms added to ME-EPI derivatives
21.0.x	December 2021	Adopt BIDS derivatives standard as the default output layout<br><br>SDCFlows major version update with new uniform API and adoption of FSL topup for PEPolar workflow (replacing AFNI 3dQwarp)<br><br>BOLD masking workflow revised to mitigate potential overcropping

Supplementary Table 2: Summary of the fMRIPrep change log from versions 21.0.x to 23.3.x
